# Cardiovascular Risk Associated with Interactions among Polymorphisms in Genes from the Renin-Angiotensin, Bradykinin, and Fibrinolytic Systems

**DOI:** 10.1371/journal.pone.0012757

**Published:** 2010-09-15

**Authors:** John P. Bentley, Folkert W. Asselbergs, Christopher S. Coffey, Patricia R. Hebert, Jason H. Moore, Hans L. Hillege, Wiek H. van Gilst

**Affiliations:** 1 Department of Pharmacy Administration, School of Pharmacy, University of Mississippi, University, Mississippi, United States of America; 2 Department of Cardiology, Division Heart & Lungs, University Medical Center Utrecht, Utrecht, The Netherlands; 3 Department of Biostatistics, College of Public Health, University of Iowa, Iowa City, Iowa, United States of America; 4 Charles E. Schmidt College of Biomedical Science, Florida Atlantic University, Boca Raton, Florida, United States of America; 5 Departments of Genetics and Community and Family Medicine, Dartmouth Medical School, Lebanon, New Hampshire, United States of America; 6 Department of Cardiology, University Medical Center Groningen, University of Groningen, Groningen, The Netherlands; 7 Department of Experimental Cardiology, University Medical Center Groningen, University of Groningen, Groningen, The Netherlands; Ohio State University Medical Center, United States of America

## Abstract

**Background:**

Vascular fibrinolytic balance is maintained primarily by interplay of tissue plasminogen activator (t-PA) and plasminogen activator inhibitor type 1 (PAI-1). Previous research has shown that polymorphisms in genes from the renin-angiotensin (RA), bradykinin, and fibrinolytic systems affect plasma concentrations of both t-PA and PAI-1 through a set of gene-gene interactions. In the present study, we extend this finding by exploring the effects of polymorphisms in genes from these systems on incident cardiovascular disease, explicitly examining two-way interactions in a large population-based study.

**Methodology/Principal Findings:**

Data from the population-based PREVEND study in Groningen, The Netherlands (n = 8,138) were analyzed. The effects of the polymorphisms and their interactions on cardiovascular events were analyzed via Cox proportional hazards models. There was no association between five of the six polymorphisms singly and risk of cardiovascular disease. There was a significant main effect for the *ACE I/D* polymorphism for both dominant and additive coding schemes. There were significant interactions between the following polymorphism pairs even after adjustment for known risk factors: *ACE I/D* & *PAI-1 4G/5G* (*p* = 0.012), *BDKRB2 C181T* & *ACE I/D* (*p* = 0.016), *BDKRB2 C58T* & *ACE I/D* (*p* = 0.025), *BDKRB2 exon 1 I/D* & *AT1R A1166C* (*p* = 0.017), and *BDKRB2 C58T* & *AT1R A1166C* (*p* = 0.015).

**Conclusions/Significance:**

This study suggests possible interactions between genes from the RA, bradykinin, and fibrinolytic systems on the risk of cardiovascular disease, extending previous research that has demonstrated that interactions among genes from these systems influence plasma concentrations of both t-PA and PAI-1. Further explorations of these interactions are needed.

## Introduction

Both tissue plasminogen activator (t-PA) and plasminogen activator inhibitor type 1 (PAI-1) are involved in thrombus formation and degradation. Vascular fibrinolytic balance is determined by the competing effects of t-PA and PAI-1 [1]. Factors that increase PAI-1 levels and decrease t-PA levels may increase the risk of thrombosis as both are risk markers for cardiovascular disease [2], [3]. There is substantial evidence that both the renin-angiotensin (RA) and bradykinin system play a role in the regulation of PAI-1 and t-PA. Previous research has shown that infusion of angiotensin II increases PAI-1 levels in both hypertensive and normotensive patients [4]. In addition, ACE-inhibitors significantly decrease PAI-1 activity in patients with a myocardial infarction [5], in hypertensive subjects [6], and in postmenopausal women [7]. Bradykinin is a potent stimulator of t-PA in the coronary circulation and this effect was augmented by ACE-inhibition [8]. In addition, we previously demonstrated that levels of PAI-1 and t-PA in a population-based sample are partly determined by genetic polymorphisms from the RA and bradykinin systems [9]. Furthermore, we found significant interactions between polymorphisms from the renin-angiotensin (RA), bradykinin, and fibrinolytic systems on the plasma concentrations of t-PA and PAI-1 in two independent studies [10], [11].

Given their role with respect to regulating vascular fibrinolytic balance, it is possible that variations in polymorphisms in genes from these systems may ultimately influence the risk of cardiovascular disease. Some, but not all, studies have shown such associations [12]–[15]. Discrepant results for each individual polymorphism may be the result of interactions with another gene or genes, i.e., each genetic variation may be associated with cardiovascular risk only in the presence of certain other genetic variation. Such gene-gene interactions may occur in the absence of independent main effects. Thus, it is possible that interactions among genes from these three systems might modify the effect of these genes on the risk of cardiovascular disease. Studies often have limited power to explore gene-gene interactions necessitating the use of large datasets. Given this and the biologic plausibility of epistatic effects of polymorphism in genes from the RA, bradykinin, and fibrinolytic systems, the purpose of the present exploratory study was to examine the effects of two-way interactions among these polymorphisms on incident cardiovascular disease in a large, population-based study.

## Methods

### Study design and participants

Data analyzed in this study were obtained from the Prevention of Renal and Vascular End-stage Disease (PREVEND) study. The PREVEND study is a large observational study currently underway in the city of Groningen, the Netherlands (started in 1997–1998). The PREVEND cohort consists of a random sample of subjects with less than 10 mg/L of urinary albumin and a selected sample of subjects with greater than 10 mg/L of urinary albumin. A total of 8,592 subjects completed the total screening program and were followed over time. Details of the study have been described previously [16]. The original study was approved by a local medical ethics committee and all participants gave written informed consent.

The combined endpoint for the present analysis was incident cardiovascular disease, defined as death from CVD (ICD-10 I00-I02, I05-I15, I20-I28, I30-I52, I60-I89, I95-I99), hospitalization for MI (ICD-10 I21, I22), percutaneous transluminal angioplasty (PCTA), or coronary artery bypass graft (CABG). The first well-defined cardiovascular event of each participant was used for analysis. Event-free survival time for each participant was defined as the period from the date of the outpatient clinic baseline assessment to the date of death, MI, PCTA, or CABG or death from any cause or until December 31, 2005, the latest date on which information regarding specific causes of death follow-up information was available.

Only subjects with a negative history of coronary artery disease (CAD) at baseline were included in the present analysis. Previous documented CAD was defined as history of myocardial infarction, revascularization procedure, or obstructive coronary artery disease prior to inclusion in the PREVEND study. A history of myocardial infarction was based on subjects' medical history, including a structured questionnaire, and information on previous CAD was complemented by review of the medical report. This approach has been used by other published reports from the PREVEND study [17]. Of the 8,592 PREVEND subjects, 453 were excluded for this reason, and one additional subject was excluded due to a missing value on the selection variable (i.e., urinary albumin concentration), leaving an analysis sample of 8,138.

Demographic and clinical information was collected for each participant at baseline. In addition, genotyping was conducted on six polymorphisms as described by Asselbergs et al. [11]. These polymorphisms (with rs numbers), classified by system, can be found in Table 1. Genotype information was unavailable for a number of participants. For example, the largest number of missing cases is for the *ACE I/D* polymorphism (missing 674 subjects or 8.3% of the analysis sample). About 89% of the subjects had complete data on all six polymorphisms (*n* = 7,260). To evaluate the potential impact of missing genotype information, six new variables were created to identify cases that had missing data on each of the polymorphisms and another variable to indicate whether a case was missing data on any of the polymorphisms (composite variable). Times to first well-defined cardiovascular event between subjects with missing data and without missing data on each of the six polymorphisms (and the composite variable) were compared using Kaplan-Meier estimates. None of the log-rank tests revealed significantly different survival curves (*df* = 1, *P*>0.50 for each of the tests). Based on these findings, for each subsequent analysis, a complete-case approach was utilized (i.e., participants who were missing genotype information were excluded). A similar approach was used by Borggreve et al. [18].

**Table 1:** Baseline characteristics of study population (n = 8,138).

Characteristic	Subcategory	Mean (SD) or %
Age (n = 8,138)		48.6 (12.5)
Body mass index (kg/m^2^) (n = 8,050)		26.0 (4.2)
Presence of diabetes (n = 7,981)		3.4%
Systolic blood pressure (mmHg) (n = 8,134)		128.7 (20.2)
Diastolic blood pressure (mmHg) (n = 8,134)		73.9 (9.8)
HDL cholesterol (mmol/L) (n = 7,940)		1.3 (0.4)
Total cholesterol (mmol/L) (n = 8,072)		5.6 (1.1)
C-reactive protein (mg/L) (n = 7,721)		2.6 (4.9)
Current smoking status (n = 8,109)		38.1%
Waist-hip ratio (n = 8,052)		0.88 (0.09)
Sex (n = 8,138)	Male	48.9%
	Female	51.1%
***Bradykinin system***		
*Bradykinin receptor B2 C181T*	*CC* (*AA*)	80.0%
*(BDKRB2 C181T)* (rs1046248) (n = 7,873)	*CT* (*Aa*)	18.7%
	*TT* (*aa*)	1.3%
*Bradykinin receptor B2 C58T*	*CC* (*AA*)	33.1%
*(BDKRB2 C58T)* (rs1799722) (n = 7,880)	*CT* (*Aa*)	48.2%
	*TT* (*aa*)	18.8%
*Bradykinin receptor B2 exon 1 insertion/deletion*	*−9/−9* (*AA*)	28.6%
*(BDKRB2 exon 1 I/D)* 1 (n = 7,796)	*−9/+9* (*Aa*)	50.7%
	*+9/+9* (*aa*)	20.7%
***Renin-angiotensin system***		
*Angiotensin II receptor type I A1166C*	*AA* (*AA*)	50.1%
*(AT1R A1166C)* (rs5186) (n = 7,740)	*AC* (*Aa*)	41.1%
	*CC* (*aa*)	8.8%
*Angiotensin converting enzyme insertion/deletion*	*ACE II* (*AA*)	24.8%
*(ACE I/D)* (rs4646994) (n = 7,464)	*ACE ID* (*Aa*)	48.3%
	*ACE DD* (*aa*)	26.9%
***Fibrinolytic system***		
*Plasminogen activator inhibitor-1 4G5G*	*5G5G* (*AA*)	21.6%
*(PAI-1 4G/5G)* (rs1799768) (n = 7,774)	*4G5G* (*Aa*)	49.1%
	*4G4G* (*aa*)	29.3%

1 The BDKRB2 exon 1 polymorphism was done with primers flanking the 9 basepair insertion/deletion site, 5′-FAM-AGCCCTTGAAAGATGAGCTGT as forward primer and 5′-CTCTGTGCTGGGACAGTTTG as reversed primer, resulting in 222 and 213 basepair products for the insertion or deletion allele, respectively.

### Statistical analysis

SAS 9.1 (SAS Institute, Cary, NC, USA) and STATA SE 9.1 (StataCorp LP, College Station, TX, USA) were used for data analysis. The effects of the polymorphisms on cardiovascular events were analyzed via Cox proportional hazards models. The proportional hazards assumption for the polymorphisms and potential confounders (i.e., demographic and clinical variables) were assessed by graphing log-log survival functions and by evaluation interactions between each variable with time. These examinations suggested that there were no substantial violations of the proportional hazards assumption.

In the screening phase of the PREVEND study, subjects with an elevated UAE were over-sampled to acquire sufficient subjects with microalbuminuria (*n* = 5,661 with UAC ≥10 mg/L and *n* = 2,477 with UAC <10 mg/L at baseline in the present analysis sample). To overcome this selection bias in the present study, design-based analyses were performed using sampling probability weights. Due to this weighting method, our conclusions can be generalized for the general population [19]. Previously published reports from the PREVEND study have implemented similar design-based analyses [20], [21]. The design-based Cox regression models were built with STATA.

As an initial step, the unadjusted, unmoderated effects of each polymorphism were examined. Prior to this analysis, all genotypes were coded (*AA* = 0, *Aa* = 1, *aa* = 2) to capture additive effects, (*AA* = 0, *Aa* or *aa* = 1) to capture dominant effects, and (*AA* or *Aa* = 0, *aa* = 1) to capture recessive effects (where *AA* = homozygous wild-type, *Aa* = heterozygous, and *aa* = homozygous variant; see Table 1 for the correspondence of *AA*, *Aa*, and *aa* to each genotype for each polymorphism).

The second step was to analyze unadjusted, two-way interaction effects for the 15 possible pairs of polymorphisms. For each pair, nine possible interaction effects were tested (additive by additive, additive by dominant, additive by recessive, dominant by additive, dominant by dominant, dominant by recessive, recessive by additive, recessive by dominant, and recessive by recessive). We used an uncorrected significance level of α = 0.10 as suggestive evidence for interaction and a more conservative significance level of α = 0.025 as strong evidence for interaction. A similar approach was used by Asselbergs et al. [11] who argue that given the exploratory and early-stage nature of their work, they were more concerned with false negatives (type II errors) than false positives (type I errors). A comparable argument can be made for the present hypothesis-generating study. For readers wishing to use more stringent criteria, *P* values for these tests are reported in supporting information tables S1a-S1f.

The third step of the analysis was to adjust the estimates by including the following demographic and clinical variables collected at baseline: sex, age, body mass index, presence of diabetes, systolic and diastolic blood pressure, HDL cholesterol, total cholesterol, c-reactive protein, current smoking status, and waist-to-hip ratio. Prior to interpretation, for pairs of polymorphisms where there were multiple significant interactions (e.g., additive-additive and dominant-recessive), we selected the model that provided the most parsimonious fit to the data using Akaike's information criterion (AIC) from the covariate-adjusted model.

Finally, because there is some evidence that the prediction of plasma t-PA/PAI-1 levels by these polymorphisms may differ by sex [9] and that the relationship between plasma concentrations of t-PA/PAI-1 and cardiovascular disease-related traits may also differ by sex [22], we evaluated whether the significant interactions differed by sex by fitting three-way interactions (sex x genotype1 x genotype2) in both the unadjusted and adjusted models.

## Results

Baseline demographic and clinical characteristics of the sample can be found in Table 1. Groups with small numbers of cases can lead to large parameter estimates and standard errors (i.e., instability of model fitting) in Cox regression. The frequency for the homozygous variant genotype for the *BDKRB2 C181T* polymorphism (*TT*) was rare (1.3%). Therefore, neither recessive coding (i.e., *TT* versus *CT* + *CC*) nor additive coding (i.e., *CC* versus *CT* versus *TT*) were used for this polymorphism in any analysis. The symbol “N/A” reflects these models in subsequent tables.

During a median follow-up of 7.52 years (range 1 day to 8.3 years), a total of *n* = 463 events were detected among the 8,138 subjects in the dataset (5.7%). Table 2 presents results of the analysis of the unadjusted, unmoderated effects of each polymorphism for each coding scheme. Five of the six polymorphisms showed non-significant effects. However, the main effect for the *ACE I/D* polymorphism was significant at the 0.05 level for the dominant (HR = 0.72, 95% CI: 0.52–0.99, *P* = 0.042) as well as the additive coding scheme (HR = 0.79, 95% CI: 0.65–0.98, *P* = 0.029). The findings suggest a smaller hazard associated with the presence of each *ACE D* allele (i.e., moving from 0 (*ACE II* genotype) to 1 (*ACE ID* genotype) to 2 (*ACE DD* genotype)). This effect is opposite of what one would expect to find based on previous research. The *ACE DD* genotype has been found to be associated with increased levels of PAI-1 in some [23], but not all [24], studies and also increased levels of angiotensin-converting enzyme (ACE) [25]. High levels of both PAI-I and ACE promote thrombosis.

**Table 2:** Univariate models for each polymorphism.

	Dominant Model^1^	Recessive Model^2^	Additive model (linear model)^3^
Gene Polymorphism	HR (*P* value)	HR (*P* value)	HR (*P* value)
*BDKRB2 C181T* (n = 7,805)	1.12 (0.510)	N/A	N/A
*BDKRB2 C58T* (n = 7,809)	0.94 (0.657)	1.11 (0.579)	1.00 (0.996)
*BDKRB2 exon 1 I/D* (n = 7,728)	1.00 (0.986)	0.76 (0.132)	0.92 (0.377)
*AT1R A1166C* (n = 7,675)	1.12 (0.443)	1.08 (0.760)	1.08 (0.458)
*ACE I/D* (n = 7,405)	**0.72 (0.042)**	0.76 (0.114)	**0.79 (0.029)**
*PAI-1 4G/5G* (n = 7,706)	0.87 (0.425)	0.88 (0.440)	0.90 (0.338)

1 *AA* = 0, *Aa* or *aa* = 1.

2 *AA* or *Aa* = 0, *aa* = 1.

3 *AA* = 0, *Aa* = 1, *aa* = 2.

For the correspondence of *AA*, *Aa*, and *aa* to each genotype for each polymorphism, see Table 1.

We found evidence for RAS-fibrinolytic and bradykinin-RAS interaction effects on incident CVD (*P* values for the statistical tests for unadjusted two-way interactions among the polymorphisms are provided supporting information tables S1a-S1f. Significant results with strong or suggestive evidence for interaction are in bold). A total of 6 gene-gene interactions were detected out of 15 possible pairs of polymorphisms. Of the 9 possible interactions for the combination of *ACE I/D* and *PAI-1 4G/5G*, 8 were significant (the *ACE I/D* x *PAI-1 4G/5G* (recessive-dominant) was the only non-significant interaction for this pair).

A summary of the 16 gene-gene interactions with strong or suggestive evidence can be found in Table 3 along with the covariate-adjusted results. The model with the lowest AIC among covariate-adjusted models within each pair of polymorphisms is noted. For one of the interactions (i.e., *AT1R A1166C* x *PAI-1 4G/5G*), it appears that adjusting for known risk factors attenuates the suggestive significance observed in the unadjusted analysis (unadjusted *P* = 0.026, adjusted *P* = 0.151). Hence, this interaction is not further explored. Statistical significance was maintained for the other five interactions following adjustment for potential confounders.

**Table 3:** Summary of the 16 significant interactions.

Interaction	Covariate unadjusted	Covariate adjusted^2^
***RAS-Fibrinolytic***		
*ACE I/D* x *PAI-1 4G/5G*		
Additive-dominant	*P* = 0.060, AIC = 5539.332	*P* = 0.060, AIC = 4536.416
dominant-additive	*P* = = 0.014, AIC = 5536.024	*P* = 0.015, AIC = 4534.768
dominant-recessive	*P* = 0.046, AIC = 5540.314	*P* = 0.106, AIC = 4541.769
dominant-dominant	*P* = 0.043, AIC = 5539.895	*P* = 0.015, AIC = 4535.571
recessive-additive	*P* = 0.054, AIC = 5543.344	*P* = 0.117, AIC = 4543.034
Additive-recessive	*P* = 0.011, AIC = 5535.290	*P* = 0.024, AIC = 4535.330
recessive-recessive	*P* = 0.033, AIC = 5543.093	*P* = 0.038, AIC = 4541.215
Additive-additive^1^	*P* = 0.007, AIC = 5532.568	*P* = 0.012, AIC = 4531.608
*AT1R A1166C* x *PAI-1 4G/5G*		
dominant-dominant^1^	*P* = 0.026, AIC = 5759.063	*P* = 0.151, AIC = 4683.674
***Bradykinin-RAS***		
*BDKRB2 C181T* x *ACE I/D*		
dominant-additive	*P* = 0.079, AIC = 5547.935	*P* = 0.106, AIC = 4541.118
dominant-recessive^1^	*P* = 0.015, AIC = 5546.962	*P* = 0.016, AIC = 4539.052
*BDKRB2 C58T* x *ACE I/D*		
recessive-dominant^1^	*P* = 0.093, AIC = 5549.522	*P* = 0.025, AIC = 4544.774
*BDKRB2 exon 1 I/D* x *AT1R A1166C*		
recessive-dominant^1^	*P* = 0.076, AIC = 5635.521	*P* = 0.028, AIC = 4577.440
recessive-additive	*P* = 0.087, AIC = 5635.938	*P* = 0.017, AIC = 4578.655
*BDKRB2 C58T* x *AT1R A1166C*		
recessive-additive^1^	*P* = 0.020, AIC = 5766.845	*P* = 0.051, AIC = 4686.709
recessive-recessive	*P* = 0.003, AIC = 5768.088	*P* = 0.015, AIC = 4693.869

1 Indicates most parsimonious model for the set of significant models within this pair of polymorphisms (model with lowest AIC among the covariate-adjusted models).

2 Covariate-adjusted models are adjusted for sex, age, body mass index, presence of diabetes, systolic and diastolic blood pressure, HDL, total cholesterol, c-reactive protein, current smoking status, and waist-to-hip ratio.

The subgroup effects of the five remaining gene-gene interactions are further explored in Table 4. The *BDKRB2 C58T* x *ACE I/D* interaction shows non-significant subgroup effects in the unadjusted model and one significant subgroup effect only after adjustment for covariates. Given these findings and the exploratory nature of this study, we do not provide further interpretations of this interaction. For the four remaining interactions, the interaction appears to be due to significant effects of one genetic variation only at certain levels of other genetic variation (Table 4). Each of these interactions is further described below.

**Table 4:** Adjusted and unadjusted hazard ratios for incident CVD for given gene polymorphism at levels of another polymorphism.

	Unadjusted HR		Adjusted HR^1^	
Interaction	(95% CI)	*P*	(95% CI)	*P*
***ACE I/D*** ** (additive** 2 **) x ** ***PAI-1 4G/5G*** ** (additive)**				
*ACE I/D* at *PAI-1 4G4G*	1.18 (0.83–1.65)	0.360	1.13 (0.79–1.62)	0.506
*ACE I/D* at *PAI-1 4G5G*	0.78 (0.64–0.96)	0.018	0.74 (0.60–0.92)	0.008
*ACE I/D* at *PAI-1 5G5G*	0.52 (0.36–0.76)	0.001	0.49 (0.32–0.75)	0.001
***BDKRB2 C181T*** ** (dominant) x ** ***ACE I/D*** ** (recessive)**				
*BDKRB2 C181T* (*TT* + *CT* versus *CC*) at *ACE DD*	0.41 (0.17–0.99)	0.049	0.52 (0.21–1.31)	0.165
*BDKRB2 C181T* (*TT* + *CT* versus *CC*) at *ACE II or ACE ID*	1.36 (0.93–1.99)	0.118	1.77 (1.20–2.61)	0.004
***BDKRB2 C58T*** ** (recessive) x ** ***ACE I/D*** ** (dominant)**				
*BDKRB2 C58T* (*TT* versus *CT* + *CC*) at *ACE II*	1.64 (0.88–3.05)	0.118	2.23 (1.21–4.09)	0.010
*BDKRB2 C58T* (*TT* versus *CT* + C*C*) at *ACE DD or ACE ID*	0.85 (0.53–1.34)	0.477	0.92 (0.57–1.48)	0.717
***BDKRB2 exon 1 I/D*** ** (recessive) x ** ***AT1R A1166C*** ** (dominant)**				
*BDKRB2 exon 1 I/D* (+9/+9 versus −9/+9 + −9/−9) at *AT1R A1166C AA*	0.52 (0.30–0.90)	0.019	0.45 (0.24–0.82)	0.009
*BDKRB2 exon 1 I/D* (+9/+9 versus −9/+9 + −9/−9) at *AT1R A1166C CC or AT1R A1166C AC*	1.01 (0.62–1.64)	0.967	1.08 (0.66–1.76)	0.770
***BDKRB2 C58T*** ** (recessive) x ** ***AT1R A1166C*** ** (additive)**				
*BDKRB2 C58T* (*TT* versus *CT* + *CC*) at *AT1R A1166C AA*	1.57 (1.00–2.46)	0.046	1.67 (1.04–2.70)	0.035
*BDKRB2 C58T* (*TT* versus *CT* + *CC*) at *AT1R A1166C AC*	0.86 (0.56–1.33)	0.498	0.98 (0.64–1.52)	0.941
*BDKRB2 C58T* (*TT* versus *CT* + *CC*) at *AT1R A1166C CC*	0.47 (0.21–1.08)	0.076	0.58 (0.25–1.35)	0.207

1 Adjusted for sex, age, body mass index, presence of diabetes, systolic and diastolic blood pressure, HDL, total cholesterol, c-reactive protein, current smoking status, and waist-to-hip ratio.

2 Additive model coding for *ACE I/D*: *II* = 0, *ID* = 1, *DD*.

Among those with at least one *PAI-1 5G* allele, there is a significantly lower hazard of cardiovascular disease associated with the presence of each *ACE D* allele (For *PAI-1 4G5G*: adjusted HR = 0.74, 95% CI: 0.60–0.92; For *PAI-1 5G5G*: adjusted HR = 0.49, 95% CI: 0.32–0.75). For those with the *PAI-1 4G4G* genotype, the effect of the *ACE I/D* polymorphism was in the opposite direction, but not statistically significant (adjusted HR = 1.13, 95% CI: 0.79–1.62).

Among those with at least one *ACE I* allele, individuals with the *BDKRB2 C181T TT* or *BDKRB2 C181T CT* genotypes had a significantly higher hazard of cardiovascular disease as compared to those who carried the *BDKRB2 C181T CC* genotype (adjusted HR = 1.77, 95% CI: 1.20–2.61). For those with the *ACE DD* genotype, the effect of the *BDKRB2 C181T* polymorphism was in the opposite direction, but was not statistically significant in the adjusted model (although it was statistically significant in the model unadjusted for covariates) (adjusted HR = 0.52, 95% CI: 0.21–1.31).

For those with the *AT1R A1166C AA* genotype, individuals with the *BDKRB2 exon 1 I/D* +9/+9 genotype had a significantly lower hazard of cardiovascular disease as compared to those who carried either the *BDKRB2 exon 1 I/D* −9/+9 or −9/−9 genotypes (adjusted HR = 0.45, 95% CI: 0.24–0.82). Among those with at least one *AT1R A1166C C* allele, the effect of the *BDKRB2 exon 1 I/D* polymorphism was in the opposite direction, but was not statistically significant (adjusted HR = 1.08, 95% CI: 0.66–1.76).

For those with the *AT1R A1166C AA* genotype, individuals with the *BDKRB2 C58T TT* or *BDKRB2 C58T CT* genotypes had a significantly higher hazard of cardiovascular disease as compared to those who carried the *BDKRB2 C58T CC* genotype (adjusted HR = 1.67, 95% CI: 1.04–2.70). Among those with at least one *AT1R A1166C C* allele, the effects of the *BDKRB2 C58T* polymorphism were in the opposite direction, but not statistically significant.

To assess whether the four significant gene-gene interactions varied by sex, three-way interaction terms were added to each model (sex x genotype1 x genotype2), along with the appropriate lower order effects (i.e., the main effects plus the following two-way interactions: genotype1 x genotype2, sex x genotype1, and sex x genotype2). For three of the four interactions, none of the three-way interactions were statistically significant at the 0.10 level of significance in the unadjusted or adjusted models. However, for the *ACE I/D* x *PAI-1 4G/5G* (additive-additive) interaction, the three-way interaction was significant in the unadjusted (*P* = 0.028) and the adjusted models (*P* = 0.028). Thus, sex may play a role in the *ACE I/D* x *PAI-1 4G/5G* interaction. Preliminary examination of the results suggests that the findings described earlier for the *ACE I/D* x *PAI-1 4G/5G* interaction may be stronger for females than for males. There was strong evidence of an *ACE I/D* x *PAI-1 4G/5G* interaction effect for females (adjusted *P* = 0.001), while the evidence was less convincing for males (adjusted *P*>0.10) (data not shown). Subsequent research is necessary to validate this finding.

## Discussion

Vascular fibrinolytic balance is maintained primarily by an interplay of t-PA and PAI-1 [1] and polymorphisms in genes from the RA, bradykinin, and fibrinolytic systems may affect plasma concentrations of both t-PA and PAI-1 through a set of gene-gene interactions [10], [11], suggesting a potential involvement of these systems in determining the risk of cardiovascular disease. The present study suggests possible interactions between the *ACE I/D* and *PAI-1 4G/5G* polymorphisms, the *BDKRB2 C181T* and *ACE I/D* polymorphisms, the *BDKRB2 exon 1 I/D* and *AT1R A1166C* polymorphisms, and the *BDKRB2 C58T* and *AT1R A1166C* polymorphisms on the risk of cardiovascular disease. The interaction between the *ACE I/D* and *PAI-1 4G/5G* polymorphisms appears to be robust to the choice of genotype coding scheme. Interestingly, several of these interactions were noted in a study of the epistatic effects of these polymorphisms on plasma t-PA and PAI-1 levels [11]. Specifically, 5 out of the 6 interactions noted in the present study were also found by Asselbergs et al. [11] to be significant in the prediction of t-PA or PAI-1 in either males or females – the exception is the *BDKRB2 C181T* x *ACE I/D* interaction).

The association between *ACE I/D* and risk of cardiovascular disease appears to be limited to those who carry the *PAI-1 5G* allele and suggests a protective effect of the *ACE D* allele. While an unexpected finding, a protective effect of *ACE DD* in the presence of the *PAI-1 5G* allele is consistent with the multifactor dimensionality reduction analysis findings of Coffey et al. [26]. In addition, other studies showed also a protective effect of the D allele on the occurrence of thrombosis [27], [28]. Interestingly, both studies found that the effect was more pronounced in men suggesting a gender interaction. Moreover, the absolute PAI-levels were lower in the *ACE DD* group in the presence of *PAI-1 5G5G*, which would be expected considering the observed protective effect in the present study (data not shown). Although not significant in the present study, there was a suggestion of increased risk of the *ACE D* allele among those with the *PAI-1 4G4G* genotype in the present study. This is consistent with previous basic science and human clinical studies. Both the *ACE DD* genotype and the *PAI-1 4G* allele are associated with increased PAI-1 levels [23], [29], [30], which promotes thrombosis and is a known risk factor for myocardial infarction.

The other three interactions involve the bradykinin and RA systems. Bradykinin plays an important role in determining t-PA levels. In addition to its role in the inflammatory process, bradykinin is a potent endothelial cell stimulant that has been shown to induce the acute release of t-PA from the endothelium through a B2 receptor mechanism [31]. In addition, ACE-inhibition augments the effects of bradykinin on the B2 receptor, which leads to t-PA release from the endothelium [32], providing a mechanistic explanation for the interaction between the bradykinin and RA systems. Moreover, genetic polymorphisms of the *RAS* and *bradykinin* genes showed epistatic effects on both PAI-1 as t-PA levels among females and males, similar to the results in the current study.

Controlling for traditional risk factors of cardiovascular disease did not lead to substantive changes in the results with respect to statistical significance. However, this does not allow us to totally rule out chance as a potential explanation of the findings. Although there are plausible biologic mechanisms to support interactions among genes from the RA, bradykinin, and fibrinolytic systems, this study used an exploratory analysis plan searching for all possible two-way interactions between six genes. Furthermore, for each possible pair of polymorphisms, we tested nine different interactions based on different genotype coding schemes. Multiple tests increase the possibility of chance associations, especially when conducting a non-replicated study. Given that these tests are correlated in the present study, it is difficult to calculate how many interactions are expected by chance. However, by making some simplifying assumptions, and noting that a total of 105 interactions were tested (nine possible interaction effects were tested for the 15 possible pairs of polymorphisms minus 30 interactions since only dominant coding was used for the *BDKRB2 C181T* polymorphism), one can estimate that under the null hypothesis, one would expect 2.6 and 10.5 interactions to be nominally significant at α = 0.025 and α = 0.10, respectively, by chance alone (as described earlier, these are the two uncorrected significance levels used in the present study). We found a total of 16 significant gene-gene interactions (*P*<0.10), most of which remained significant after adjusting for known risk factors. The findings of significant interactions between genes from the RA, bradykinin, and fibrinolytic systems on the risk of cardiovascular disease warrant further exploration, including attempting to replicate these results in other populations with other study designs.

It is important to recognize that cardiovascular disease is a relatively infrequent event in this cohort due to the young age (mean 48.6 years at baseline) and gender composition (51% female). The onset of symptoms is age related and additional cardiovascular events are expected to be observed in the PREVEND cohort as more time passes. In addition, the penetrance of the alleles may also be age related. Future analyses should examine whether these potential interactions remain as additional events become available and the population grows older.

Furthermore, because this study was limited to the investigation of potential two-way interactions, future studies should consider higher-order interactions using a multifactor dimensionality reduction (MDR) type analysis [33], [34]. Such analyses have revealed the presence of gene-gene interactions in the absence of statistically significant independent main effects and also may assist in confirming the findings of the current exploratory study. Future research should also explore the role of sex in moderating these interactions. Finally, although this study was intended to evaluate possible underlying mechanisms and not to suggest the use of these gene polymorphisms as diagnostic tools, it may be of some interest to explore whether the significant gene-gene interactions found in the present study can improve the predictive ability of known models [35], [36].

In summary, this study suggests possible interplay between genes from the RA, bradykinin, and fibrinolytic systems on the risk of cardiovascular disease, extending previous research that has demonstrated that interactions among genes from these systems influence plasma concentrations of both t-PA and PAI-1.

## Supporting Information

Table S1 Table S1a - S1f. P values for the unadjusted first-order gene-gene interactions are reported in supporting information tables S1a-S1f.
(0.09 MB DOC)
