# Supplementary material for: Cardiovascular Risk Associated with Interactions among Polymorphisms in Genes from the Renin-Angiotensin, Bradykinin, and Fibrinolytic Systems
Source: PLoS One. 2010 Sep 15;5(9):e12757. doi: 10.1371/journal.pone.0012757 (PMC2939877; doi:10.1371/journal.pone.0012757)
Supplement: Table S1 — a - S1f. P values for the unadjusted first-order gene-gene interactions are reported in supporting information tables S1a-S1f. (0.09 MB DOC) [file pone.0012757.s001.doc]

**Table S1a. Unadjusted first-order gene-gene interactions – *P* value for the interaction term^1^ (Dominant by Additive)**

| **Polymorphism** | *BDKRB2 C181T* | *BDKRB2*  *C58T* | *BDKRB2*  *exon 1 I/D* | *AT1R*  *A1166C* | *ACE I/D* | *PAI-1*  *4G/5G* |
| --- | --- | --- | --- | --- | --- | --- |
| *BDKRB2 C181T* | - | 0.886 | 0.899 | 0.918 | **0.079** | 0.718 |
| *BDKRB2 C58T* | N/A | - | 0.852 | 0.395 | 0.491 | 0.879 |
| *BDKRB2 exon 1 I/D* | N/A | 0.668 | - | 0.508 | 0.187 | 0.108 |
| *AT1R A1166C* | N/A | 0.730 | 0.148 | - | 0.682 | 0.244 |
| *ACE I/D* | N/A | 0.246 | 0.227 | 0.542 | - | **0.014** |
| *PAI-1 4G/5G* | N/A | 0.799 | 0.501 | 0.107 | **0.060** | - |

^1^Model included main effects for both gene polymorphisms and the first-order interaction. *P* values for interaction effects between genotypes from Cox proportional hazards models. The different genotype codings are listed. For example, the first *P* value listed is 0.886 for the interaction between *BDKRB2 C181T* (dominant model) and *BDKRB2 C58T* (additive model).

**Table S1b. Unadjusted first-order gene-gene interactions – *P* value for the interaction term^1^ (Dominant by Recessive)**

| **Polymorphism** | *BDKRB2 C181T* | *BDKRB2*  *C58T* | *BDKRB2*  *exon 1 I/D* | *AT1R*  *A1166C* | *ACE I/D* | *PAI-1*  *4G/5G* |
| --- | --- | --- | --- | --- | --- | --- |
| *BDKRB2 C181T* | - | 0.777 | 0.947 | 0.699 | **0.015** | 0.808 |
| *BDKRB2 C58T* | N/A | - | 0.685 | 0.627 | 0.462 | 0.922 |
| *BDKRB2 exon 1 I/D* | N/A | 0.647 | - | 0.785 | 0.355 | 0.217 |
| *AT1R A1166C* | N/A | 0.107 | **0.076** | - | 0.305 | 0.801 |
| *ACE I/D* | N/A | **0.093** | 0.546 | 0.398 | - | **0.046** |
| *PAI-1 4G/5G* | N/A | 0.554 | 0.696 | 0.562 | 0.298 | - |

^1^Model included main effects for both gene polymorphisms and the first-order interaction. *P* values for interaction effects between genotypes from Cox proportional hazards models. The different genotype codings are listed. For example, the first *P* value listed is 0.777 for the interaction between *BDKRB2 C181T* (dominant model) and *BDKRB2 C58T* (recessive model).

**Table S1c. Unadjusted first-order gene-gene interactions – *P* value for the interaction term^1^ (Dominant by Dominant)**

| **Polymorphism** | *BDKRB2 C181T* | *BDKRB2*  *C58T* | *BDKRB2*  *exon 1 I/D* | *AT1R*  *A1166C* | *ACE I/D* | *PAI-1*  *4G/5G* |
| --- | --- | --- | --- | --- | --- | --- |
| *BDKRB2 C181T* | - |  |  |  |  |  |
| *BDKRB2 C58T* | 0.898 | - |  |  |  |  |
| *BDKRB2 exon 1 I/D* | 0.739 | 0.851 | - |  |  |  |
| *AT1R A1166C* | 0.947 | 0.417 | 0.489 | - |  |  |
| *ACE I/D* | 0.696 | 0.656 | 0.228 | 0.798 | - |  |
| *PAI-1 4G/5G* | 0.376 | 0.891 | 0.172 | **0.026** | **0.043** | - |

^1^Model included main effects for both gene polymorphisms and the first-order interaction. *P* values for interaction effects between genotypes from Cox proportional hazards models. The different genotype codings are listed. For example, the first *P* value listed is 0.898 for the interaction between *BDKRB2 C58T* (dominant model) and *BDKRB2 C181T* (dominant model).

**Table S1d. Unadjusted first-order gene-gene interactions – *P* value for the interaction term^1^ (Recessive by Additive)**

| **Polymorphism** | *BDKRB2 C181T* | *BDKRB2*  *C58T* | *BDKRB2*  *exon 1 I/D* | *AT1R*  *A1166C* | *ACE I/D* | *PAI-1*  *4G/5G* |
| --- | --- | --- | --- | --- | --- | --- |
| *BDKRB2 C181T* | - | N/A | N/A | N/A | N/A | N/A |
| *BDKRB2 C58T* | N/A | - | 0.686 | **0.020** | 0.449 | 0.953 |
| *BDKRB2 exon 1 I/D* | N/A | 0.563 | - | **0.087** | 0.235 | 0.750 |
| *AT1R A1166C* | N/A | 0.305 | 0.572 | - | 0.761 | 0.834 |
| *ACE I/D* | N/A | 0.815 | 0.153 | 0.296 | - | **0.054** |
| *PAI-1 4G/5G* | N/A | 0.751 | 0.457 | 0.861 | **0.011** | - |

^1^Model included main effects for both gene polymorphisms and the first-order interaction. *P* values for interaction effects between genotypes from Cox proportional hazards models. The different genotype codings are listed. For example, the first *P* value listed is 0.563 for the interaction between *BDKRB2 exon 1 I/D* (recessive model) and *BDKRB2 C58T* (additive model).

**Table S1e. Unadjusted first-order gene-gene interactions – *P* value for the interaction term^1^ (Recessive by Recessive)**

| **Polymorphism** | *BDKRB2 C181T* | *BDKRB2*  *C58T* | *BDKRB2*  *exon 1 I/D* | *AT1R*  *A1166C* | *ACE I/D* | *PAI-1*  *4G/5G* |
| --- | --- | --- | --- | --- | --- | --- |
| *BDKRB2 C181T* | - |  |  |  |  |  |
| *BDKRB2 C58T* | N/A | - |  |  |  |  |
| *BDKRB2 exon 1 I/D* | N/A | 0.658 | - |  |  |  |
| *AT1R A1166C* | N/A | **0.003** | 0.489 | - |  |  |
| *ACE I/D* | N/A | 0.687 | 0.162 | 0.566 | - |  |
| *PAI-1 4G/5G* | N/A | 0.650 | 0.878 | 0.382 | **0.033** | - |

^1^Model included main effects for both gene polymorphisms and the first-order interaction. *P* values for interaction effects between genotypes from Cox proportional hazards models. The different genotype codings are listed. For example, the first *P* value listed is 0.658 for the interaction between *BDKRB2 exon 1 I/D* (recessive) and *BDKRB2 C58T* (recessive model).

**Table S1f. Unadjusted first-order gene-gene interactions – *P* value for the interaction term^1^ (Additive by Additive)**

| **Polymorphism** | *BDKRB2 C181T* | *BDKRB2*  *C58T* | *BDKRB2*  *exon 1 I/D* | *AT1R*  *A1166C* | *ACE I/D* | *PAI-1*  *4G/5G* |
| --- | --- | --- | --- | --- | --- | --- |
| *BDKRB2 C181T* | - |  |  |  |  |  |
| *BDKRB2 C58T* | N/A | - |  |  |  |  |
| *BDKRB2 exon 1 I/D* | N/A | 0.665 | - |  |  |  |
| *AT1R A1166C* | N/A | 0.509 | 0.174 | - |  |  |
| *ACE I/D* | N/A | 0.396 | 0.109 | 0.845 | - |  |
| *PAI-1 4G/5G* | N/A | 0.957 | 0.386 | 0.279 | **0.007** | - |

^1^Model included main effects for both gene polymorphisms and the first-order interaction. *P* values for interaction effects between genotypes from Cox proportional hazards models. The different genotype codings are listed. For example, the first *P* value listed is 0.665 for the interaction between *BDKRB2 exon 1 I/D* (additive model) and *BDKRB2 C58T* (additive model).
